# Atlas of Clinical Medicine

**Published:** 1892-09

**Authors:** 


*Atlas of Clinical Medicine.* By BYROM BRAMWELL, M.D. Volume I. Part IV. Edinburgh: T. and A. CONSTABLE. 1892.

It is enough to say that this number of Dr. Byrom Bramwell's book is fully up to the level of excellence of the preceding parts. It contains a full and lucid account of small-pox in its various forms, considered mainly from the clinical point of view, and illustrated by four plates. Then a unique case of globulinuria is described, in which, amongst other peculiar features, crystals of globulin were deposited spontaneously in the urine. Dr. Nöel Paton contributes the results of an exhaustive research into the chemical characters of this crystalline deposit. The notes of three fresh cases of Friedreich's Ataxia, observed by the author, are given, and four admirable plates, three of which are examples of melancholia, and one of mania, bring to a close the first volume of this work, remarkable alike for the beauty of its illustrations and the ability of the accompanying letterpress.

